# The Role of Competence in Nursing: A Cross‐Sectional Study Among Croatian Undergraduate and Graduate Students: Empirical Research Quantitative

**DOI:** 10.1002/nop2.70285

**Published:** 2025-08-08

**Authors:** Kata Ivanišević, Goran Hauser, Sandra Bošković

**Affiliations:** ^1^ Faculty of Health Study University of Rijeka Rijeka Croatia; ^2^ Faculty of Medicine University of Rijeka Rijeka Croatia

**Keywords:** competency assessment, nursing, nursing curriculum, nursing education, professional competence

## Abstract

**Aim:**

To assess the professional competencies of Croatian undergraduate and graduate nursing students using the Nurse Professional Competence (NPC) scale and to identify factors influencing these competencies.

**Design:**

Cross‐sectional, correlational study.

**Methods:**

The study was conducted between April and July 2022. A stratified random sample of 750 nursing students was selected from four higher education institutions in Croatia. The data was collected by means of an electronic survey using the Croatian language adapted version of the Nurse Professional Competence scale. The scale consists of 35 items in six domains, which are rated on a 7‐point Likert scale. Internal consistency was measured using Cronbach's *α*. The data were analysed using SPSS version 25, with both parametric and non‐parametric tests applied depending on the data distribution.

**Results:**

A total of 750 nursing students with an average age of 31 years (SD = 10.54) participated in the study. The internal consistency of the six competency factors ranged from Cronbach's *α* = 0.75 (Medical and Technical Care) to *α* = 0.90 (Nursing Care, Care Pedagogics). The highest self‐assessed competence was in Value‐Based Nursing Care (M = 6.05, SD = 0.75) and the lowest in Medical and Technical Care (M = 4.96, SD = 0.88). Significant differences were found between the institutions in various areas of competence, with students at the University of Split achieving better results in several areas. Age and level of education were found to be significant predictors of competence in several areas.

**Patient or Public Contribution:**

No input from patients or the public. This study focused solely on nursing students' self‐assessment in an educational setting.

## 1 Introduction

Technological advances, changing healthcare demands and evolving patient expectations have brought new roles and responsibilities for nurses. Nursing as a profession is undergoing constant change, driven by the increasing complexity of patient care and the rising expectations of healthcare systems. From both a professional and clinical perspective, it is important that nurses acquire the necessary skills to provide high‐quality care (Keshk et al. 2018; Falcó‐Pegueroles et al. 2021). In this context, competence in nursing proves to be a fundamental concept.

Competence is crucial for ensuring high‐quality, ethical and safe patient care (Kendall‐Gallagher and Blegen 2009). It is recognised as an integral part of professional standards and is defined as the ability to integrate knowledge, skills, attitudes and values in specific clinical contexts (Meretoja et al. 2004). Furthermore, competence is closely linked to the professional identity of nurses (Fitzgerald 2020; Fitzgerald and Clukey 2021). Key components of competence include clinical skills, patient safety and the ability to perform professional tasks effectively (Kirwan et al. 2019; Al‐Moteri 2020; Mlambo et al. 2021; Vázquez‐Calatayud et al. 2021; Innes and Calleja 2018).

Research on nursing competence has gained considerable momentum in recent decades (Blazun et al. 2015), but competence remains an abstract concept that is difficult to define and measure. Consequently, clear definitions are needed to develop valid and reliable assessment tools (Smith 2012). Research from the 2000s and various conceptual analyses underline the complexity of the competence concept (Axley 2008; Scott Tilley 2008; Garside and Nhemachena 2013). More recent studies on competence measurement aim to clarify the core aspects of the concept. Reliable and valid instruments are essential, and the best instrument must be selected on the basis of thorough methodological studies and systematic reviews (Mokkink et al. 2010).

A widely used instrument for assessing general nursing competence is the Nurse Professional Competence Scale, which has been translated into several languages. Its validity and reliability have been confirmed in various cultural contexts, including Croatia. Competent nurses are essential to provide high‐quality healthcare. In the 21st century, the role of nurses has expanded significantly and this development is likely to continue. Within the Croatian healthcare system, the importance of nurses is increasingly recognised, highlighting the need to monitor and assess their competencies at both undergraduate and graduate levels.

Despite the global attention to nursing competence, there is limited evidence on how effectively Croatian nursing education prepares students across all core areas of competence. This represents a significant research gap, particularly in understanding institutional differences and influencing factors such as age and education level.

Nursing competence is not only a foundational professional attribute but also a determinant of patient safety, healthcare quality and readiness for clinical practice. As such, it represents a variable of high importance for health systems and society.

In light of these considerations, there is a clear need to conduct empirical research focused on Croatian nursing students in order to assess current competence levels and identify areas for educational improvement.

The aim of this study is to assess the professional competencies of undergraduate and graduate nursing students using the Nurse Professional Competence Scale, and to examine the factors that influence these competencies.

## 2 Material and Methods

### 2.1 Objectives

The primary aim of this study is to assess the professional competencies of undergraduate and graduate nursing students in Croatia using the NPC scale. The secondary aim is to identify factors that influence these competencies and to suggest changes to the curriculum based on the results.

### 2.2 Study Design

A correlational cross‐sectional design was used to collect data from a random sample of 750 nursing students. The sample included undergraduate and graduate students who were about to graduate from four major nursing institutions in Croatia: the Faculty of Health Studies in Rijeka, the Croatian Catholic University in Zagreb, the Faculty of Dental Medicine and Health in Osijek and the University Department of Health Studies in Split.

### 2.3 Participants

The study sample consisted of nursing students from the aforementioned institutions. Only students who completed all six competency factors in the self‐assessment questionnaire were included in the analysis. Students who were not enrolled in nursing programmes or who submitted incomplete questionnaires were excluded.

Random selection ensured that nursing students were represented throughout Croatia, in both urban and regional areas. However, the results may not be fully generalisable to all Croatian nursing students, especially those from smaller institutions that were not included in this study. Future studies should include a broader sample to increase representativeness.

### 2.4 Instrument

Data were collected using a descriptive characteristics form and the Nurse Professional Competence Scale. The descriptive form was used to collect demographic data such as age, gender, education level and institution. The NPC scale, developed by Swedish researchers in collaboration with the World Health Organization (WHO), is based on the competency requirements for registered nurses in Sweden (The National Board of Health and Welfare 2005; Nilsson et al. 2014). The scale measures nursing competence using 35 statements divided into six domains. Each item is rated on a 7‐point Likert scale, where 1 indicates ‘To a very low degree’ and 7 indicates ‘To a very high degree’. The score for each domain is calculated as the arithmetic mean of the responses within that domain.

– Nursing Care (5 items).
– Value‐Based Nursing Care (5 items).
– Medical and Technical Care (6 items).
– Care Pedagogics (5 items).
– Documentation and Administration of Nursing Care (8 items).
– Development, Leadership and Organisation of Nursing Care (6 items).

The NPC scale has shown high internal consistency and reliability, with Cronbach's alpha values between 0.71 and 0.86 (Nilsson et al. 2018). For this study, the scale was adapted to the Croatian context, and confirmatory factor analysis (CFA) showed satisfactory goodness‐of‐fit indices (RMSEA = 0.085, CFI = 0.84) with significant factor loadings (*p* < 0.05). Cronbach's alpha for the six factors was between 0.76 and 0.92, confirming the reliability of the adjusted instrument (Ivanišević et al. 2022).

### 2.5 Data Collection

The survey was conducted electronically. Nursing students received an invitation by email explaining the purpose, objectives and participation rights of the study. By clicking on a link provided, participants were able to access and complete the online questionnaire. The survey was open from April to July 2022, with reminder emails sent every 2 weeks to ensure an adequate response rate.

The data was stored securely on the 1 ka.si web server, which was password protected. Only the principal investigator had access to the database, and individual results were anonymised.

### 2.6 Data Analysis

The data were analysed using SPSS for Windows (version 25) [SPSS Inc., Chicago, IL, USA]. Parametric and non‐parametric tests were performed depending on the distribution of the variables. Continuous data were presented as arithmetic means and standard deviations, while categorical and nominal data were presented as absolute numbers and frequencies. The chi‐square test (*χ* ^2^) was used to assess differences in categorical variables, and the *t*‐test was used to assess differences in quantitative data. Statistical significance was set at *p* < 0.05.

### 2.7 Ethical Considerations

Ethical approval was granted by the Biomedical Research Ethics Committees of all participating institutions. All participants were fully informed about the purpose and aims of the study. Participation was voluntary and confidentiality was ensured by anonymising all data collected.

## 3 Results

The analysis included 750 respondents who completed all six scales for self‐assessment of professional competencies. The descriptive statistics provide general information about the respondents, expressed as frequencies (*f*) and percentages (%). The average age of the respondents was 31 years (M = 31.01, SD = 10.54).

### 3.1 Internal Consistency

Descriptive statistics at item level and reliability coefficients for internal consistency (Cronbach's alpha) were calculated for each competency scale. All six factors showed satisfactory reliability, with the lowest reliability coefficient for the Medical and Technical Care factor (Cronbach's *α* = 0.75) and the highest for Nursing Care (Cronbach's *α* = 0.90) and care pedagogics (Cronbach's *α* = 0.90).

### 3.2 Average Values

The lowest self‐assessed competence values were given for the factor Medical and Technical Care (M = 4.96, SD = 0.88; after excluding extreme values: M = 4.97, SD = 0.86) and the factor Development, Leadership and Organisation of Nursing Care (M = 5.09, SD = 1.05). Although these values are the lowest, they still reflect relatively high self‐assessed competencies.

The highest average value was determined for the Value‐Based Nursing Care factor (M = 6.05, SD = 0.75; after excluding extreme values, M = 6.06, SD = 0.72), indicating a strong self‐assessment of proficiency in this domain (Table 1).

**TABLE 1:** Descriptive statistics of the factors with and without outliers.

	Mean (M)	Standard deviation (SD)	Mean without outliers	SD without outliers
Medical and Technical Care	4.96	0.88	4.97	0.86
Development Leadership and Organisation of Nursing Care	5.09	1.05	—	—
Value‐Based Nursing Care	6.05	0.75	6.06	0.72

*Note:* Descriptive statistics are given as mean values (M) and standard deviations (SD). If outlier‐adjusted values are available, these are shown in the columns ‘Mean without outliers’ and ‘SD without outliers’. A em dash (‘—’) means that no outlier‐adjusted values are available.

### 3.3 Correlation Between the Competence Factors

Pearson correlation coefficients (*r*) were calculated to examine the relationships between the self‐assessed competencies in the different areas. All correlations were statistically significant (*p* < 0.001) and ranged from 0.50 (between Value‐Based Nursing Care and Development, Leadership and Organisation of Nursing Care) to 0.78 (between Care Pedagogics and Documentation and Administration of Nursing Care) (Table 2).

**TABLE 2:** Correlations (*r*) between the competence factors.

	1	2	3	4	5	6
(1) Nursing Care	1.00					
(2) Value‐Based Nursing Care	0.62***	1.00				
(3) Medical and Technical Care	0.60***	0.60***	1.00			
(4) Care Pedagogics	0.62***	0.64***	0.72** *	1.00		
(5) Documentation and Administration of Nursing Care	0.67***	0.66***	0.73** *	0.78** *	1.00	
(6) Development Leadership and Organisation of Nursing Care	0.54***	0.50***	0.64** *	0.64** *	0.71** *	1.0 0

*Note:* Pearson correlation coefficients (*r*) are shown. All correlations are statistically significant (***p < 0.001).

### 3.4 Differences in Competencies by Institution

A one‐way analysis of variance (ANOVA) was performed to examine the differences in professional competencies between graduates of the four institutions: (1) Faculty of Health Studies Rijeka, (2) Croatian Catholic University Zagreb, (3) Faculty of Dental Medicine and Health Osijek and (4) University Department of Health Studies Split. Significant differences were found in several areas (Table 3):

– Nursing Care: a significant institutional effect was found (*F* [3, 740] = 3.92, *p* < 0.01, *ɳ*p^2^ = 0.02). The post hoc Bonferroni test showed that graduates from Split (M = 5.92, SD = 0.82) performed significantly better than those from Zagreb (M = 5.55, SD = 0.89) and Osijek (M = 5.60, SD = 0.90). No significant differences were found between the other groups.
– Medical and Technical Care: a significant institutional effect was observed (*F* [3, 744] = 5.15, *p* < 0.01, *ɳ*p^2^ = 0.02). Graduates from Split (M = 5.26, SD = 0.73) performed better than those from Osijek (M = 4.85, SD = 0.84) and Rijeka (M = 4.95, SD = 0.88).
– Documentation and Administration of Nursing Care: a significant institutional effect was found (*F* [3, 744] = 2.70, *p* < 0.05, *ɳ*p^2^ = 0.01). Graduates from Split (M = 5.91, SD = 0.80) performed better than graduates from Osijek (M = 5.62, SD = 0.88).
– Development, Leadership and Organisation of Nursing Care: a significant institutional effect was found (*F* [3, 746] = 11.92, *p* < 0.001, *ɳ*p^2^ = 0.05). Graduates from Split (M = 5.60, SD = 1.03) had higher scores than graduates from Osijek (M = 4.86, SD = 1.02), Rijeka (M = 5.06, SD = 1.00) and Zagreb (M = 5.19, SD = 1.06). In addition, graduates from Zagreb performed better than those from Osijek.

**TABLE 3:** ANOVA results: Differences in competency scores by institution.

	Institution	M	SD	*F* (df)	*p*	Post hoc comparisons (Bonferroni)
Nursing Care	Split	5.92	0.82	3.92 (3740)	< 0.01	Split > Zagreb, Osijek
	Zagreb	5.55	0.89			
	Osijek	5.60	0.90			
	Rijeka	5.70	0.83			
Medical and Technical Care	Split	5.26	0.73	5.15 (3744)	< 0.01	Split > Osijek, Rijeka
	Zagreb	5.05	0.84			
	Osijek	4.85	0.84			
	Rijeka	4.95	0.88			
Documentation and Administration of Nursing Care	Split	5.91	0.80	2.70 (3744)	< 0.05	Split > Osijek
	Zagreb	5.70	0.82			
	Osijek	5.62	0.88			
	Rijeka	5.68	0.85			
Development Leadership and Organisation of Nursing Care	Split	5.60	1.03	11.92 (3746)	< 0.001	Split > Osijek, Rijeka, Zagreb; Zagreb > Osijek
	Zagreb	5.19	1.06			
	Osijek	4.86	1.02			
	Rijeka	5.06	1.00			

*Note:* *F*‐statistics and *p*‐values are given for ANOVA tests. Post hoc comparisons indicate significant differences between institutions.

### 3.5 Predictors of Competence

Regression analyses showed that age and educational level explained part of the variance in several competency areas (Table 4):

– Nursing Care: age and education level explained 10.5% of the variance (*R* ^2^ = 0.105, *F* [2, 741] = 43.51, *p* < 0.001), with older age and higher education level associated with better competencies.
– Value‐Based Nursing Care: Educational level explained 2.8% of the variance (*R* ^2^ = 0.028, *F* [2, 744] = 10.89, *p* < 0.001), with higher education leading to better competencies.
– Medical and Technical Care: Age and education level explained 10.0% of the variance (*R* ^2^ = 0.100, *F* [2, 745] = 41.30, *p* < 0.001). Older and more highly educated respondents indicated higher competencies.
– Care Pedagogics: Age and education level explained 6.5% of the variance (*R* ^2^ = 0.065, *F* [2, 743] = 25.92, *p* < 0.001), with higher competencies associated with higher age and education.
– Documentation and Administration of Nursing Care: age and education level explained 9.0% of the variance (*R* ^2^ = 0.090, *F* [2, 745] = 36.79, *p* < 0.001).
– Development, Leadership and Organisation of Nursing Care: Age and education level explained 16.0% of the variance (*R* ^2^ = 0.160, *F* [2, 747] = 71.07, *p* < 0.001), with older and more educated respondents demonstrating higher competencies.

**TABLE 4:** Regression analysis: Influence of age and education on competencies.

	Predictor	*β*	SE	*t*	*p*	*R* ^2^	*F* (df)
Nursing Care	Age	0.22	0.04	5.74	< 0.001	0.105	43.51 (2, 741)
	Education Level	0.26	0.03	6.67	< 0.001		
Value‐Based Nursing Care	Education Level	0.17	0.05	4.18	< 0.001	0.028	10.89 (2, 744)
Medical and Technical Care	Age	0.19	0.03	5.32	< 0.001	0.100	41.30 (2, 745)
	Education Level	0.28	0.04	7.13	< 0.001		
Care Pedagogics	Age	0.17	0.03	4.91	< 0.001	0.065	25.92 (2, 743)
	Education Level	0.22	0.04	5.39	< 0.001		

Abbreviations: *β*, standardised beta coefficient; *p*, significance level; SE, standard error; *t*, *t*‐value.

### 3.6 Age Differences by Institution

Due to unequal variance, a Welch test was performed (Levene test, *F* (3, 746) = 16.02, *p* < 0.001), which revealed significant age differences between the institutions (Welch *F* (3, 230) = 27.32, *p* < 0.001) (Table 5). Graduates from Split (M = 37.95, SD = 11.57) were significantly older than graduates from the other institutions. Graduates from Osijek (M = 27.05, SD = 8.57) were significantly younger compared to the other institutions. No significant differences were found between Rijeka (M = 31.92, SD = 10.32) and Zagreb (M = 31.39, SD = 10.53).

**TABLE 5:** Age differences by institution (Welch's test).

Institution	Mean age (M)	Standard deviation (SD)	Welch *F* (df)	*p*	Post hoc comparisons (Games‐Howell)
Split	37.95	11.57	Welch *F* (3, 230) = 27.32	< 0.001	Split > Osijek, Zagreb, Rijeka
Osijek	27.05	8.57			Osijek < Split, Zagreb, Rijeka
Zagreb	—	—			
Rijeka	—	—			

*Note:* Due to a violation of the homogeneity of variance, the Welch test was used (Levene test: *F* (3, 746) = 16.02, *p* < 0.001).

### 3.7 Differences in Educational Level

A chi‐square test revealed a significant correlation between the institution and the level of education completed (*χ* ^2^ = 26.97, df = 3, *N* = 750, *p* < 0.001), indicating an unequal representation of undergraduates with Bachelor's and Master's degrees in the different institutions (Table 6).

**TABLE 6:** Chi‐square test: Educational level by institution.

Institution	Undergraduate (%)	Graduate (%)	*χ* ^2^ (df)	*p*
Split	65.2	34.8	26.97 (3)	< 0.001
Osijek	78.5	21.5	—	—
Zagreb	72.0	28.0	—	—
Rijeka	69.5	30.5	—	—

*Note:* The *χ* ^2^ (df) and *p*‐values are only given for significant tests (in this case for Split). For institutions where no significant effect was found, the values for the test and the *p*‐value are marked with ‘—’ for clarity.

### 3.8 Analysis With Control for Age and Education

When controlling for age and level of education, there was no significant effect of the institution on Nursing Care, Value‐Based Nursing Care, Medical and Technical Care, Care Pedagogics or Documentation and Administration of Nursing Care. However, a small but significant institutional effect was observed for Development, Leadership and Organisation of Nursing Care (*F* (3, 744) = 3.62, *p* < 0.05, *ɳ*p^2^ = 0.01). The graduates from Split performed better in this area than those from Osijek.

## 4 Discussion

The assessment of nurses' professional competencies is crucial for improving both nursing education and the overall quality of healthcare (Nilsson et al. 2015). In the Croatian context, this study provides valuable insights into the competence levels of student nurses and identifies areas for improvement. The use of the Nursing Professional Competence Scale allowed us to measure self‐assessed competencies, which is crucial for identifying gaps in clinical practice and assessing the quality of nursing education. The results suggest that while Croatian nursing students have a high level of self‐confidence in their professional competencies, issues related to the professional identity of nurses, as highlighted by Charette et al. (2020), remain relevant.

The Nursing profession has changed significantly over the last three decades due to the increasing complexity of healthcare and the increasing demand for high quality and safe patient care (Church 2016). This evolution, driven by scientific research and evidence‐based practice, has led to the integration of increasingly complex procedures into nursing care. Consequently, nursing education must ensure that students acquire not only the necessary knowledge and skills, but also the competencies required to meet these evolving demands (Kahriman and Öztürk 2016). Competency‐based education is the cornerstone of effective and quality healthcare (Aiken et al. 2014), which is why it is crucial to assess these competencies in different healthcare and educational systems to promote harmonisation and transparency worldwide.

The results of this study are consistent with the European standards of Directives 2005/36/EC and 2013/55/EU, as the self‐assessments of Croatian nursing students indicate a high level of compliance with these standards. Interestingly, the lowest self‐assessments were in the areas of ‘Medical and Technical Care’ and ‘Development, Leadership and Organisation of Nursing Care’, while the highest scores were in the area of ‘Value‐Based Nursing Care’. This reflects a strong alignment between the national curriculum and the professional values enshrined in the European Framework for nursing. However, the gaps identified in technical and leadership point to the need for more practical training and interdisciplinary engagement. These findings emphasise the importance of value‐based education that promotes ethical and compassionate care (Petrova et al. 2006).

Cross‐cultural differences in the assessment of competencies suggest that educational systems and healthcare environments play a crucial role in the expression of nursing competencies. For example, studies from industrialised countries such as Australia and Sweden report higher levels of self‐assessed competencies among nurses (Cowin et al. 2008; Gardulf et al. 2016), while lower levels of self‐assessment have been observed in developing countries (Nabirye et al. 2011). This discrepancy illustrates the influence of socioeconomic conditions, legislation and cultural factors on the development and perception of nursing competencies.

Competency‐based nursing education should also consider the broader context of disaster preparedness and emergency management, particularly in relation to global challenges such as heatwaves and emergency situations. For example, the study by Khademipour et al. (2017) on crowd simulations and critical density points in emergencies provides valuable insights into how nurses can respond in high‐pressure scenarios, directly contributing to the development of crisis management competencies. Incorporating such simulations into nursing education can better prepare students for real‐world emergencies.

Similarly, Farokhzadian et al. (2024) explore the challenges nurses face in disaster response and emphasise the importance of leadership, rapid decision‐making and effective teamwork in high‐stress environments. These aspects of nursing competence are essential for an effective disaster response and are often insufficiently covered in traditional nursing curricula.

In addition, Kiarsi et al. (2023) examine the need for nurses to adapt to environmental health hazards such as heatwaves. As climate change increases the frequency and severity of these events, it is becoming increasingly important to prepare nurses for environmental health crises. These studies highlight the need to integrate a broader range of competencies into nursing education to ensure nurses are prepared to manage both routine and crisis situations effectively. These broader requirements underline the importance of institutional support, innovative teaching methods and opportunities for applied learning, which were also observed as influencing factors in our study.

The study also found significant differences between Croatian institutions, particularly in areas such as ‘Nursing’, ‘Medical and Technical Care’ and ‘Documentation and Administration of Nursing Care’. These differences can be attributed to factors such as the quality of teaching resources, the integration of innovative teaching methods and partnerships with healthcare institutions that provide students with practical experience. Simulation‐based learning has been shown to be an effective strategy for improving clinical decision‐making and reducing errors in practice (Eyikara and Baykara 2017), emphasising the need for wider implementation of such methods in Croatian nursing education. In addition, the level of academic and emotional support provided by institutions can significantly influence competence development (Utvær et al. 2022).

The ‘Medical and Technical Care’ factor in the NPS emphasises the need for students to develop competencies in medical procedures, the use of technology and adherence to medical protocols. Differences in this competency are likely to be influenced by different opportunities for clinical placements and practical experiences in different institutions. Studies in developing countries such as Malawi have shown that partnerships between educational institutions and health facilities can enhance the acquisition of practical skills (Bvumbwe 2016). In addition, student motivation and attitude play an important role in competency development (Rafii et al. 2019), which emphasises the importance of fostering a motivating educational environment.

No significant differences were found between the groups in terms of leadership and organisational skills. This is consistent with the view that leadership skills education is fundamental and applicable at all levels of nursing education (Curtis et al. 2011). The integration of management competencies into nursing curricula is crucial to the development of nurses who are able to lead teams and contribute to the improvement of healthcare.

Age and level of education have been found to have a positive impact on competencies in the areas of ‘Nursing Care’, ‘Medical and Technical Care’, ‘Value‐Based Nursing Care’ and ‘Documentation and Administration of Nursing Care’. Older students may be better able to deal with complex situations and make independent decisions in the clinical setting due to their greater life and work experience (Allan et al. 2016). In addition, a higher level of education correlates with a deeper understanding of ethical principles and values that are critical to values‐based nursing practice (Petrova et al. 2006).

## 5 Limitations

This study has several limitations that should be considered when interpreting the results. First, the study relies on self‐report, which introduces the possibility of bias. Participants may have over‐ or underestimated their competencies due to social desirability or lack of self‐awareness, which could affect the accuracy of the results. Self‐assessment is inherently subjective and may not fully reflect the actual level of clinical competence.

Second, the cross‐sectional design of the study provides a snapshot of students' competencies at a particular point in time. While this approach provides valuable insights, it does not take into account how these competencies evolve over the course of students' educational or professional practice. A longitudinal study would be necessary to track changes in competencies over time.

Thirdly, the generalisability of the results is limited. Although the sample was drawn from four large nursing institutions in Croatia, the results may not be fully representative of all nursing students in Croatia, especially those from smaller or less represented institutions. Consequently, the conclusions may not be transferable to all Croatian nursing programmes.

Fourth, the study excluded participants who did not complete all sections of the competency questionnaire, which may have led to selection bias. Students who were less engaged or struggled with the survey may be underrepresented, resulting in a sample that reflects a higher level of competence than is present in the wider population.

Fifth, the study focuses solely on self‐assessed competencies without including objective measures such as clinical assessments or evaluations from instructors and supervisors. While self‐assessment provides important insights into students' perceptions of their abilities, the lack of external validation limits the ability to confirm this self‐assessment with actual clinical performance.

## 6 Conclusion and Recommendations

This study provides important insights into the self‐assessed professional competencies of nursing students in Croatia and identifies both strengths and areas for improvement. The results suggest that while Croatian nursing students generally report a high level of professional competence, notable differences exist between institutions and specific domains—particularly in Medical and Technical Care, where lower self‐assessments were observed. These findings indicate a need for targeted interventions to enhance competence in this area.

The results underscore the importance of further improving the quality of nursing education in Croatia, especially through the integration of simulation‐based learning, ongoing professional development for nursing educators and regular curriculum updates. These measures could contribute to greater harmonisation between institutions, reduce competence gaps and better align nursing education with the demands of modern clinical practice.

Future research should focus on incorporating objective measures of competence, such as clinical performance evaluations or practical skill assessments, to complement self‐reported data. Longitudinal studies would also provide valuable insights into how competencies develop over time, particularly during clinical placements and early professional practice. Additionally, studies examining the impact of specific educational interventions—such as simulation‐based learning or mentoring programmes—could help identify best practices for competence development in nursing education.

## Author Contributions

K.I., G.H. and S.B. drafted the study design. K.I. was involved in the data collection. All authors were involved in the quality management, analysis and interpretation of the data. All authors were involved in the first and last draft of the manuscript and approved it for publication.

## Conflicts of Interest

The authors declare no conflicts of interest.

## Data Availability

The data that support the findings of this study are available on request from the corresponding author. The data are not publicly available due to privacy or ethical restrictions.

## References

[nop270285-bib-0001] Aiken, L. H. , D. M. Sloane , L. Bruyneel , et al. 2014. “Nurse Staffing and Education and Hospital Mortality in Nine European Countries: A Retrospective Observational Study.” Lancet 383, no. 9931: 1824–1830. 24581683 10.1016/S0140-6736(13)62631-8 PMC4035380

[nop270285-bib-0002] Allan, B. A. , K. L. Autin , and R. D. Duffy . 2016. “Self‐Determination and Meaningful Work: Exploring Socioeconomic Constraints.” Frontiers in Psychology 7: 71. 26869970 10.3389/fpsyg.2016.00071 PMC4735399

[nop270285-bib-0003] Al‐Moteri, M. 2020. “Entrustable Professional Activities in Nursing: A Concept Analysis.” International Journal of Nursing Sciences 7, no. 3: 277–284. 32817849 10.1016/j.ijnss.2020.06.009 PMC7424159

[nop270285-bib-0004] Axley, L. 2008. “Competency: A Concept Analysis.” Nursing Forum 43, no. 4: 214–222. 19076465 10.1111/j.1744-6198.2008.00115.x

[nop270285-bib-0005] Blazun, H. , P. Kokol , and J. Vosner . 2015. “Research Literature Production on Nursing Competences From 1981 Till 2012: A Bibliometric Snapshot.” Nurse Education in Practice 15, no. 5: 359–365. 25616510 10.1016/j.nedt.2015.01.002

[nop270285-bib-0006] Bvumbwe, T. 2016. “Enhancing Nursing Education via Academic–Clinical Partnership: An Integrative Review.” International Journal of Nursing Sciences 3, no. 3: 314–322.

[nop270285-bib-0008] Charette, M. , L. G. McKenna , M.‐A. Maheu‐Cadotte , M.‐F. Deschênes , L. Ha , and S. Merisier . 2020. “Measurement Properties of Scales Assessing New Graduate Nurses' Clinical Competence: A Systematic Review of Psychometric Properties.” International Journal of Nursing Studies 110: 103734. 32810719 10.1016/j.ijnurstu.2020.103734

[nop270285-bib-0009] Church, C. D. 2016. “Defining Competence in Nursing and Its Relevance to Quality Care.” Journal for Nurses in Professional Development 32, no. 5: E9–E14. 27648912 10.1097/NND.0000000000000289

[nop270285-bib-0010] Cowin, L. S. , C. Hengstberger‐Sims , S. C. Eagar , L. Gregory , S. Andrew , and J. Rolley . 2008. “Competency Measurements: Testing Convergent Validity for Two Measures.” Journal of Advanced Nursing 64, no. 3: 272–277. 18990106 10.1111/j.1365-2648.2008.04774.x

[nop270285-bib-0011] Curtis, E. A. , F. K. Sheerin , and J. de Vries . 2011. “Developing Leadership in Nursing: The Impact of Education and Training.” British Journal of Nursing 20, no. 6: 344–352. 21471889 10.12968/bjon.2011.20.6.344

[nop270285-bib-0012] Eyikara, E. , and Z. G. Baykara . 2017. “The Importance of Simulation in Nursing Education.” World Journal of Educational Technology 9, no. 1: 2–7.

[nop270285-bib-0013] Falcó‐Pegueroles, A. , D. Rodríguez‐Martín , S. Ramos‐Pozón , and E. Zuriguel‐Pérez . 2021. “Critical Thinking in Nursing Clinical Practice, Education, and Research: From Attitudes to Virtue.” Nursing Philosophy 22, no. 1: e12332. 33029860 10.1111/nup.12332

[nop270285-bib-0014] Farokhzadian, J. , P. Mangolian Shahrbabaki , N. Memaryan , and F. Abazari . 2024. “Nurses' Challenges for Disaster Response: A Qualitative Study.” BMC Emergency Medicine 24, no. 1: 12. 38172759 10.1186/s12873-023-00921-8 PMC10765941

[nop270285-bib-0015] Fitzgerald, A. 2020. “Professional Identity: A Concept Analysis.” Nursing Forum 55, no. 3: 447–472. 32249453 10.1111/nuf.12450

[nop270285-bib-0016] Fitzgerald, A. , and L. Clukey . 2021. “Professional Identity in Graduating Nursing Students.” Journal of Nursing Education 60, no. 2: 74–80. 33528577 10.3928/01484834-20210120-04

[nop270285-bib-0017] Gardulf, A. , J. Nilsson , J. Florin , J. Leksell , M. Lepp , and C. Lindholm . 2016. “The Nurse Professional Competence (NPC) Scale: Self‐Reported Competence Among Nursing Students on the Point of Graduation.” Nurse Education Today 36: 165–171. 26586256 10.1016/j.nedt.2015.09.013

[nop270285-bib-0018] Garside, J. , and J. Nhemachena . 2013. “A Concept Analysis of Competence and Its Transition in Nursing.” Nurse Education Today 33, no. 5: 541–545. 22257583 10.1016/j.nedt.2011.12.007

[nop270285-bib-0019] Innes, T. , and P. Calleja . 2018. “Transition Support for New Graduate and Novice Nurses in Critical Care Settings: An Integrative Review of the Literature.” Nurse Education in Practice 30: 62–72. 29571106 10.1016/j.nepr.2018.03.001

[nop270285-bib-0020] Ivanišević, K. , R. Kosić , S. Bošković , and M. Bukvić . 2022. “Implementation of the Nurse Professional Competence Scale in the Republic of Croatia.” SAGE Open 12, no. 3: 1–8.

[nop270285-bib-0021] Kahriman, İ. , and H. Öztürk . 2016. “Evaluating Medical Errors Made by Nurses During Their Diagnosis, Treatment, and Care Practices.” Journal of Clinical Nursing 25, no. 19–20: 2884–2894. 27335283 10.1111/jocn.13341

[nop270285-bib-0022] Kendall‐Gallagher, D. , and M. A. Blegen . 2009. “Competence and Certification of Registered Nurses and Safety of Patients in Intensive Care Units.” American Journal of Critical Care 18, no. 2: 106–116. 19255100 10.4037/ajcc2009487 PMC2717746

[nop270285-bib-0023] Keshk, L. I. , S. Qalawa , and N. A. Ibrahim . 2018. “Effectiveness of an Educational Program Regarding the Nursing Process on Acquiring Advanced Skills Among Internship Nursing Students.” International Journal of Nursing Studies 5, no. 2: 32–44.

[nop270285-bib-0024] Khademipour, G. , N. Nakhaee , S. M. S. Anari , H. Farahmandnia , and H. Rezaei . 2017. “Crowd Simulations and Determining the Critical Density Point of Emergency Situations.” Disaster Medicine and Public Health Preparedness 11, no. 6: 674–680. 28554341 10.1017/dmp.2017.7

[nop270285-bib-0025] Kiarsi, M. , M. Amiresmaili , M. R. Mahmoodi , and R. Dehnavieh . 2023. “Heat Waves and Adaptation: A Global Systematic Review.” Journal of Thermal Biology 114: 103623. 10.1016/j.jtherbio.2023.103588 37499408

[nop270285-bib-0026] Kirwan, M. , O. Riklikiene , J. Gotlib , et al. 2019. “Regulation and Current Status of Patient Safety Content in Pre‐Registration Nurse Education in 27 Countries: Findings From the RANCARE COST Action Project.” Nurse Education in Practice 37: 132–140. 31153130 10.1016/j.nepr.2019.04.013

[nop270285-bib-0029] Meretoja, R. , H. Isoaho , and H. Leino‐Kilpi . 2004. “Nurse Competence Scale: Development and Psychometric Testing.” Journal of Advanced Nursing 47, no. 2: 124–133. 15196186 10.1111/j.1365-2648.2004.03071.x

[nop270285-bib-0030] Mlambo, M. , C. Silén , and C. McGrath . 2021. “Lifelong Learning and Nurses' Continuing Professional Development: A Metasynthesis of the Literature.” BMC Nursing 20, no. 1: 1–13. 33853599 10.1186/s12912-021-00579-2 PMC8045269

[nop270285-bib-0031] Mokkink, L. B. , C. B. Terwee , D. L. Knol , et al. 2010. “The COSMIN Checklist for Evaluating the Methodological Quality of Studies on Measurement Properties: A Clarification of Its Content.” BMC Medical Research Methodology 10: 22. 20298572 10.1186/1471-2288-10-22 PMC2848183

[nop270285-bib-0032] Nabirye, R. C. , K. C. Brown , E. R. Pryor , and E. H. Maples . 2011. “Occupational Stress, Job Satisfaction, and Job Performance Among Hospital Nurses in Kampala, Uganda.” Journal of Nursing Management 19, no. 6: 760–768. 21899629 10.1111/j.1365-2834.2011.01240.x

[nop270285-bib-0034] Nilsson, J. , M. Engström , J. Florin , A. Gardulf , and M. Carlsson . 2018. “A Short Version of the Nurse Professional Competence Scale for Measuring Nurses' Self‐Reported Competence.” Nurse Education Today 71: 233–239. 30321851 10.1016/j.nedt.2018.09.028

[nop270285-bib-0035] Nilsson, J. , A. Gardulf , and M. Lepp . 2015. “Process of Translation and Adaptation of the Nurse Professional Competence (NPC) Scale.” Journal of Nursing Education and Practice 6, no. 1: 100.

[nop270285-bib-0036] Nilsson, J. , E. Johansson , A.‐C. Egmar , J. Florin , J. Leksell , and M. Lepp . 2014. “Development and Validation of a New Tool Measuring Nurses' Self‐Reported Professional Competence: The Nurse Professional Competence (NPC) Scale.” Nurse Education Today 34, no. 4: 574–580. 23938092 10.1016/j.nedt.2013.07.016

[nop270285-bib-0037] Petrova, M. , J. Dale , and B. K. Fulford . 2006. “Values‐Based Practice in Primary Care: Easing the Tensions Between Individual Values, Ethical Principles, and Best Evidence.” British Journal of General Practice 56, no. 530: 703–709. PMC1876638 16954004

[nop270285-bib-0038] Rafii, F. , M. Saeedi , and S. Parvizy . 2019. “Academic Motivation in Nursing Students: A Hybrid Concept Analysis.” Iranian Journal of Nursing and Midwifery Research 24, no. 5: 315–322. 31516515 10.4103/ijnmr.IJNMR_177_18 PMC6714123

[nop270285-bib-0039] Scott Tilley, D. D. 2008. “Competency in Nursing: A Concept Analysis.” Journal of Continuing Education in Nursing 39, no. 2: 58–64. 18323142 10.3928/00220124-20080201-12

[nop270285-bib-0040] Smith, S. A. 2012. “Nurse Competence: A Concept Analysis.” International Journal of Nursing Knowledge 23, no. 3: 172–182. 23043658 10.1111/j.2047-3095.2012.01225.x

[nop270285-bib-0041] The National Board of Health and Welfare . 2005. “Competence Requirements for Registered Nurses.” Stockholm, Sweden: The National Board of Health and Welfare.

[nop270285-bib-0042] Utvær, B. K. , H. Torbergsen , T. E. Paulsby , and G. Haugan . 2022. “Nursing Students' Emotional State and Perceived Competence During the COVID‐19 Pandemic: The Vital Role of Teacher and Peer Support.” Frontiers in Psychology 12: 793304. 35153918 10.3389/fpsyg.2021.793304 PMC8830307

[nop270285-bib-0043] Vázquez‐Calatayud, M. , B. Errasti‐Ibarrondo , and A. Choperena . 2021. “Nurses' Continuing Professional Development: A Systematic Literature Review.” Nurse Education in Practice 50: 102963. 33422973 10.1016/j.nepr.2020.102963

